# Single-institution experience with ipilimumab in advanced melanoma patients in the compassionate use setting

**DOI:** 10.1002/cncr.24951

**Published:** 2010-04-01

**Authors:** Geoffrey Y Ku, Jianda Yuan, David B Page, Sebastian E A Schroeder, Katherine S Panageas, Richard D Carvajal, Paul B Chapman, Gary K Schwartz, James P Allison, Jedd D Wolchok

**Affiliations:** 1 Ludwig Center for Cancer Immunotherapy, Memorial Sloan-Kettering Cancer CenterNew York, New York; 2 Department of Medicine, Memorial Sloan-Kettering Cancer CenterNew York, New York; 3 Department of Epidemiology and Biostatistics, Memorial Sloan-Kettering Cancer CenterNew York, New York; 4 Melanoma/Sarcoma Service, Department of Medicine, Memorial Sloan-Kettering Cancer CenterNew York, New York; 5 Department of Immunology, Memorial Sloan-Kettering Cancer CenterNew York, New York; 6 Howard Hughes Medical InstituteChevy Chase, Maryland

**Keywords:** ipilimumab, melanoma, lymphocyte, compassionate use, trial

## Abstract

**BACKGROUND:**

Ipilimumab is a monoclonal antibody that antagonizes cytotoxic T lymphocyte antigen-4, a negative regulator of the immune system. The authors report on advanced refractory melanoma patients treated in a compassionate use trial of ipilimumab at the Memorial Sloan-Kettering Cancer Center.

**METHODS:**

Patients with advanced refractory melanoma were treated in a compassionate use trial with ipilimumab 10 mg/kg every 3 weeks for 4 doses. Those with evidence of clinical benefit at Week 24 (complete response [CR], partial response [PR], or stable disease [SD]) then received ipilimumab every 12 weeks.

**RESULTS:**

A total of 53 patients were enrolled, with 51 evaluable. Grade 3/4 immune-related adverse events were noted in 29% of patients, with the most common immune-related adverse events being pruritus (43%), rash (37%), and diarrhea (33%). On the basis of immune-related response criteria, the response rate (CR + PR) was 12% (95% confidence interval [CI], 5%-25%), whereas 29% had SD (95% CI, 18%-44%). The median progression-free survival was 2.6 months (95% CI, 2.3-5.2 months), whereas the median overall survival (OS) was 7.2 months (95% CI, 4.0-13.3 months). Patients with an absolute lymphocyte count (ALC) ≥1000/μL after 2 ipilimumab treatments (Week 7) had a significantly improved clinical benefit rate (51% vs 0%; *P* = .01) and median OS (11.9 vs 1.4 months; *P* < .001) compared with those with an ALC <1000/μL.

**CONCLUSIONS:**

The results confirm that ipilimumab is clinically active in patients with advanced refractory melanoma. The ALC after 2 ipilimumab treatments appears to correlate with clinical benefit and OS, and should be prospectively validated. Cancer 2010. © 2010 American Cancer Society.

This description of 51 patients with advanced, treatment-refractory melanoma who were enrolled in a compassionate use trial of ipilimumab at Memorial Sloan-Kettering Cancer Center confirms that ipilimumab is active in this disease setting. In addition, the results suggest that the absolute lymphocyte count after 2 ipilimumab treatments (at Week 7) highly correlates with the rate of clinical benefit at Week 24 and overall survival.

Melanoma is 1 of several cancers whose incidence has increased in the past several decades.^1^ In the metastatic setting, therapy is toxic and relatively ineffective.^2–4^ Most trials have reported objective responses in <15% of patients, which tend to be short-lived. In fact, a clear survival benefit for chemotherapy has yet to be demonstrated. The notable exception is treatment with high-dose interleukin-2 (IL-2), which has a similarly low response rate and serious toxicity but does lead to durable complete responses in a small subset of patients. As such, more effective therapy is urgently needed.

Cytotoxic T lymphocyte antigen-4 (CTLA-4) is a coinhibitory molecule expressed by activated T cells and a subset of regulatory T cells.^5–7^ CTLA-4 is of primary importance in maintaining immune homeostasis by down-regulating T-cell signaling to inhibit the CD28-B7 costimulatory pathway, limiting T-cell responses, and contributing to tolerance to self-antigens.^8^, ^9^ Therefore, blockade of CTLA-4 is thought to prevent down-regulation of T cells and can potentiate immune responses against antigens expressed on tumor cells.^10–13^

Ipilimumab is a fully human immunoglobulin G_1_ monoclonal antibody that blocks CTLA-4. In several phase 1 and 2 trials, it has been found to produce objective and durable tumor responses in several tumor types, notably melanoma.^14^, ^15^ A group of mechanism-based side effects, termed immune-related adverse events, occur in patients treated with CTLA-4–blocking antibodies. The induction of this organ-specific inflammation underscores the importance of CTLA-4 in restraining the immune system under normal circumstances. The immune-related adverse events can be controlled using corticosteroids, according to simple algorithms. Interestingly, the frequency of clinical benefit has been observed to be higher in patients having immune-related adverse events, and the use of corticosteroids and other immunosuppressive medications does not interfere with sustained tumor immunity.^16^

In this article, we report on 51 evaluable patients with advanced melanoma treated in a compassionate use trial of ipilimumab at Memorial Sloan-Kettering Cancer Center (MSKCC). These were heavily pretreated patients who did not qualify for other clinical trials and were expected to have a poor prognosis. In addition to communicating a large single-institution experience with ipilimumab, which largely corroborates findings of multi-institutional trials, we also propose that the absolute lymphocyte count (ALC) after the first 2 treatments may be a useful biomarker to identify patients who are unlikely to benefit from ipilimumab therapy. Given the occurrence of immune-related adverse events in a significant number of patients and the sometimes long time interval required to obtain clinical benefit, a simple biomarker for identification of patients with low chance for benefit represents an important goal.

## MATERIALS AND METHODS

### Eligibility Criteria

Eligibility criteria included unresectable stage III and IV melanoma, with all histology confirmed at MSKCC. Patients had experienced progression of disease (PD) or intolerance to at least 1 prior systemic therapy (except for ocular primary tumor patients, who were required to have local control of their disease), with the last therapy administered ≥28 days before trial entry (with the exception of palliative radiotherapy). All patients were ≥18 years old and were required to have essentially normal bone marrow and organ function and an Eastern Cooperative Oncology Group (ECOG) performance status of ≤2. Patients with primary ocular or mucosal melanomas were eligible, as were those with brain metastases. Exclusion criteria included prior therapy with ipilimumab or eligibility for another ongoing trial of ipilimumab. The protocol was reviewed by the MSKCC Institutional Review Board (IRB), and all patients gave informed consent. Additional blood samples were obtained for immune function correlative assays under a separate IRB-approved procurement protocol.

### Treatment Plan

Ipilimumab (MDX-010) was provided by Bristol-Myers Squibb (Plainsboro, NJ). During the induction phase, it was administered at 10 mg/kg intravenously over 90 minutes every 3 weeks for 4 doses (on Weeks 1, 4, 7, and 10). Patients completed the induction phase unless they developed clear clinical deterioration or unacceptable toxicity (refractory grade ≥3 immune-related adverse event).

After induction ipilimumab, patients were re-evaluated at Weeks 12 and 24. Those without unacceptable toxicity and with evidence of clinical benefit at Week 24 (defined as complete response [CR] or partial response [PR] or stable disease [SD]) received maintenance ipilimumab 10 mg/kg every 12 weeks. Patients continued on therapy until PD, death or unacceptable toxicity occurred. Patients off therapy continued to be observed until death or until they were lost to follow-up.

### Evaluation at Baseline and During Treatment

Pretreatment evaluations included a complete history and physical examination, routine laboratory testing, and imaging of measurable disease. Before each ipilimumab administration, patients underwent repeat laboratory testing and were monitored for toxicity, which was graded according to National Cancer Institute Common Terminology Criteria for Adverse Events (version 3.0).

Patients underwent repeat radiographic imaging at Weeks 12 and 24 and before each subsequent dose of maintenance ipilimumab. Because responses to ipilimumab may follow an atypical pattern from cytotoxic chemotherapy, recently proposed immune-related (immune-related) response criteria were used for clinical decision-making.^17^, ^18^ These criteria represent an amendment to the modified World Health Organization (WHO) criteria so that the appearance of new lesions does not automatically constitute PD. Instead, the total tumor burden is calculated by summation of the product of the perpendicular diameters of new and previously measurable lesions. An immune-related CR occurs when all measurable disease disappears, an immune-related PR occurs when there has been >50% but <100% decrease in tumor burden, immune-related SD occurs if there has been ≤50% decrease or ≤25% increase in tumor burden, and immune-related PD occurs if the tumor burden increases by >25%.

### Statistical Analysis

Any patient who received at least 1 ipilimumab treatment and had at least 1 follow-up evaluation was eligible for analysis. As this was a compassionate use trial, there was no specific target accrual. Univariate analyses of clinical characteristics and outcomes, including response proportion (CR + PR) and clinical benefit rate (CR + PR + SD) at Week 24, were assessed using the chi-square test. Kaplan-Meier survival distributions were estimated to assess progression-free survival (PFS) and overall survival (OS). The log-rank test was used for comparison of survival distributions.

To determine whether the ALC after the first and second ipilimumab treatments, respectively, was associated with improved survival, a landmark analysis was performed in which OS was defined as the time from 3 and 6 weeks, respectively, after treatment start (approximately the time of each post-treatment complete blood count [CBC]) to the date of death or last follow-up. This was done to correct for patients who died before they underwent repeat CBC measurements.^19^ A Cox proportional hazards model was fit to adjust for baseline lactate dehydrogenase (LDH) levels when further examining the association of ALC on OS.

## RESULTS

### Demographics

From October 2007 through September 2008, 53 patients have been enrolled. Two patients are unevaluable; 1 patient was lost to follow-up after receiving 1 dose of ipilimumab, whereas the other patient received chemotherapy between ipilimumab doses. Patient demographics are summarized in Table 1.

**Table 1:** Patient Demographics (n = 53)

Age, y	
Median	62
Range	38–86
Sex	
Men	34 (64%)
Women	19 (36%)
ECOG performance status	
0	16 (30%)
1	29 (55%)
2	8 (15%)
Stage	
IIIC	1 (2%)
IV	52 (98%)
Primary site	
Cutaneous	42 (79%)
Ocular	5 (9%)
Mucosal	3 (6%)
Unknown	3 (6%)
LDH	
≤ULN (200 U/L)	23 (43%)
1.1 to ≤2× ULN	13 (25%)
>2× ULN	17 (32%)
Prior therapy	
Radiotherapy	21 (40%)
Cytotoxic chemotherapy	49 (93%)
Biologic therapy	
Adjuvant IFN-α (adjuvant)	9 (17%)
High-dose IL-2 (metastatic disease)	8 (15%)
Vaccine therapy	4 (7%)
Others (eg, small molecule inhibitors)	23 (43%)
No. of prior systemic therapies	
Median	2
Range	0–6

ECOG indicates Eastern Cooperative Oncology Group; LDH, lactate dehydrogenase; ULN, upper limit of normal; IFN-α, interferon-α; IL-2, interleukin-2.

The median age of patients was 62 years (range, 38–86 years). Approximately 64% were men, and 85% had an ECOG status of 0 to 1. Patients had relatively advanced disease as assessed by baseline LDH; 32% had a baseline LDH >2× the upper limit of normal.

Most patients were heavily pretreated; the median number of prior systemic therapies was 2 (range, 0–6 therapies). Approximately 93% had received prior cytotoxic chemotherapy (primarily temozolomide-based regimens), and 43% had also received therapy with investigational agents, such as small molecule inhibitors (eg, imatinib, sorafenib). Approximately 17% and 15%, respectively, of patients had received adjuvant interferon-α and high-dose IL-2 in the metastatic setting, whereas 7% had received prior vaccine therapy in either the adjuvant or metastatic setting.

### Toxicity

There were no treatment-related deaths, although 8 of 51 (16%) evaluable patients died within 30 days of their last dose of ipilimumab. Toxicities seen on this trial were largely consistent with the known toxicities of anti–CTLA-4 therapies in a population with advanced cancer. All immune-related adverse events, significant grade 1 to 4 toxicities noted in >20% of patients are listed in Table 2.

**Table 2:** Toxicities (n = 51)^a^

Toxicity	Grade (% of Patients)				
1	2	3	4
Adrenal insufficiency	—	1 (2)	1 (2)	—
Anemia	28 (55)	14 (27)	4 (8)	1 (2)
Colitis	—	1 (2)	3 (6)	1 (2)
Confusion	—	—	3 (6)	1 (2)
Conjunctivitis	—	2 (4)	—	—
Dehydration	—	—	3 (6)	—
Diarrhea	6 (12)	3 (6)	8 (16)	—
Dyspnea	9 (18)	4 (8)	4 (8)	1 (2)
Fatigue	17 (33)	9 (18)	4 (8)	—
Hypothyroidism	—	1 (2)	—	—
Increased ALT	9 (18)	5 (10)	2 (4)	2 (4)
Increased AST	11 (22)	4 (8)	2 (4)	2 (4)
Increased bilirubin	—	—	2 (4)	2 (4)
Increased lipase	1 (2)	—	—	1 (2)
Infection	—	—	3 (6)	2 (4)
Leukopenia	10 (20)	1 (2)	—	1 (2)
Lymphopenia	6 (12)	9 (18)	9 (18)	—
Nausea/vomiting	15 (29)	4 (8)	2 (4)	—
Neutropenia	—	—	—	1 (2)
Pain	10 (20)	8 (16)	2 (4)	—
Pruritis	18 (35)	4 (8)	—	—
Rash	13 (25)	5 (10)	1 (2)	—
Thrombosis	—	—	1 (2)	1 (2)
Thrombocytopenia	1 (2)	2 (4)	2 (4)	1 (2)

ALT indicates alanine aminotransferase; AST, aspartate aminotransferase.

aToxicities were graded according to National Cancer Institute Common Terminology Criteria for Adverse Events (version 3.0).

Treatment-related grade 3 to 4 hematologic toxicity was noted only in 1 patient (2%), a 47-year-old man who had previously developed pancytopenia on temozolomide therapy, considered an idiosyncratic reaction. After receiving the third dose of ipilimumab, he developed grade 4 neutropenia and thrombocytopenia and grade 2 anemia. Bone marrow biopsy revealed a hypercellular marrow with an infiltrate of lymphocytes and plasma cells, along with granuloma formation. He received filgrastim, intravenous methylprednisolone, intravenous immunoglobulin, infliximab, and finally cyclosporine. His blood counts eventually recovered but, presumably because of the 4-week period of neutropenia and immunosuppression therapy, he subsequently developed Fournier gangrene, requiring extensive surgical debridement. Fortunately, he has since recovered from all toxicities.

In terms of nonhematologic toxicities, a constellation of immune-related adverse events related to the inhibition of negative regulation by CTLA-4 was observed. The most common immune-related adverse events were grade 1 to 2 pruritus in 22 (43%) patients and rash in 19 (37%, 1 grade 3) patients. Diarrhea was noted in 17 (33%) patients, with 8 (16%) patients experiencing grade 3 diarrhea. Five (10%) of these patients were also found to have colitis by colonoscopy. Other serious immune-related adverse events included grade 3 to 4 aspartate aminotransferase/alanine aminotransferase elevation secondary to noninfectious hepatitis in 4 (8%) patients, adrenal insufficiency in 2 patients (4%, 1 grade 2 with evidence of hypophysitis on magnetic resonance imaging and 1 grade 3) patients, grade 2 conjunctivitis in 2 patients, and grade 4 lipase elevation secondary to pancreatitis in 1 patient. One patient developed grade 2 hypothyroidism without other evidence of endocrine dysfunction that may have been treatment related.

Overall, 15 of 51 (29%) patients developed 1 or more grade 3 to 4 immune-related adverse events. Seven (14%) patients discontinued therapy because of immune-related adverse events.

### Clinical Responses

On the basis of the immune-related response criteria, we noted a pattern of response that was atypical for that of cytotoxic chemotherapy. One (2%) patient experienced an immune-related PD before experiencing immune-related SD. Twelve (24%) patients experienced prolonged immune-related SD as their best response, with a median time to progression of 8.4 months.

**Figure 1:**
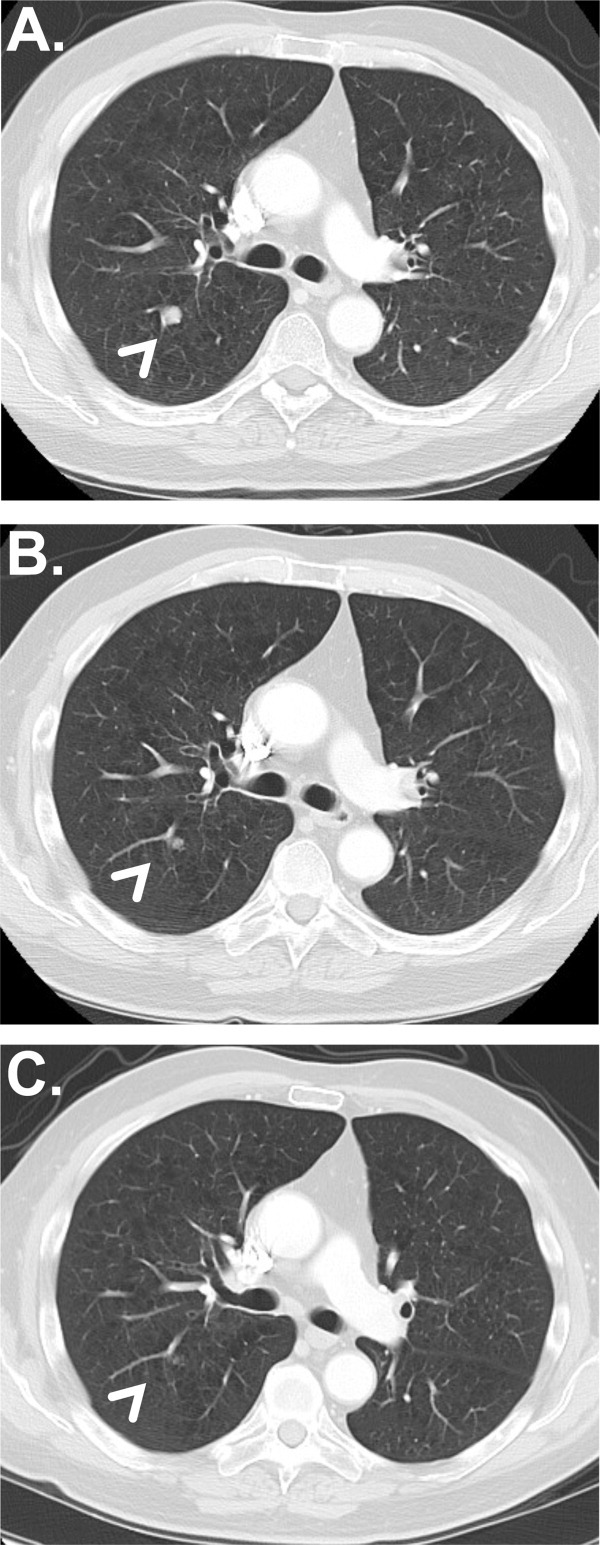
Radiographic images of a patient with a complete response are shown. Representative images were obtained (A) at baseline, (B) after completing induction with 4 treatments of ipilimumab every 3 weeks, and (C) at 9 months (before the second dose of maintenance ipilimumab). The patient was alive at the time of last follow-up without evidence of disease recurrence, 11.2 months after the initiation of protocol therapy.

The objective response rate (RR) was 12% (4 immune-related CRs and 2 immune-related PRs; 95% confidence interval [CI], 5%-25%). Of the patients with immune-related CRs, 1 patient had completed radiation therapy just before study entry and experienced a CR after ipilimumab therapy in lesions that were within and outside of the radiation field. Another patient underwent resection of the only site of residual metastatic disease after experiencing a CR at other metastatic sites and has since maintained a CR. A third patient (who experienced grade 4 hematologic toxicity as described above) underwent resection of pelvic lymph nodes that comprised the only site of residual metastatic disease (at the time of a colostomy reversal) and was found to have achieved a pathologic CR. Characteristics of these patients are presented in Table 3; representative radiographic images of a patient with a CR are shown in Figure 1.

**Table 3:** Clinical Characteristics of Patients With Objective Responses

Age, y/Sex	Baseline LDH	No. of Prior Therapies	Disease Sites	Best Response	Time to Response, mo	Response Duration, mo	OS, mo
76/Man	113	3	Lung, soft tissue	irCR^a^	3.5	10.2+	13.7+
78/Woman	160	3	Lung, soft tissue, bone	irPR	2.4	2.8	8.3
62/Man	92	4	Soft tissue	irCR^b^	5.2	6.2+	11.4+
66/Man	121	2	Lung, soft tissue, lymph node	irCR	5.6	5.6+	11.2+
62/Man	171	3	Lung, lymph node	irPR	4.3	6+	10.3+
47/Man	210	1	Lymph node	irCR^c^	2.6	4.4+	7.0+

LDH indicates lactate dehydrogenase; OS, overall survival; irCR, immune-related complete response; irPR, immune-related partial response.

a This patient experienced a complete response to ipilimumab and surgery.

b This patient received radiotherapy prior to study entry.

c This patient was found to have achieved a pathologic complete response when the only metastatic site was resected.

Fifteen (29%; 95% CI, 18%-44%) patients experienced immune-related SD as their best response, whereas the remaining 30 patients (59%) experienced immune-related PD/death. As the decision to continue maintenance ipilimumab was based on an assessment at Week 24, we also calculated the clinical benefit rate at Week 24, which was 33% (95% CI, 21%-48%).

Although patients with and without high-grade immune-related adverse events experienced objective response, there appeared to be a correlation between the development of grade 3 to 4 immune-related adverse events and clinical response. Patients with grade 3 to 4 immune-related adverse events had a significantly higher clinical benefit rate at Week 24 (9 of 15 [60%] patients vs 8 of 36 [22%] patients; *P* < .01) compared with those with grade ≤2 immune-related adverse events. There was also a borderline significant trend toward an increased objective RR in patients with grade 3 to 4 immune-related adverse events (4 of 15 [27%] vs 2 of 36 [6%]; *P* < .05).

### Survival

The median PFS of all 51 patients was 2.6 months (95% CI, 2.3–5.2 months). The median OS was 7.2 months (95% CI, 4.0–13.3 months). There were no significant differences in OS when patients were stratified by known prognostic factors in melanoma: baseline LDH, number of prior systemic therapies, and cutaneous versus mucosal/ocular primary tumors.

### Biomarker Evaluation: ALC

**Figure 2:**
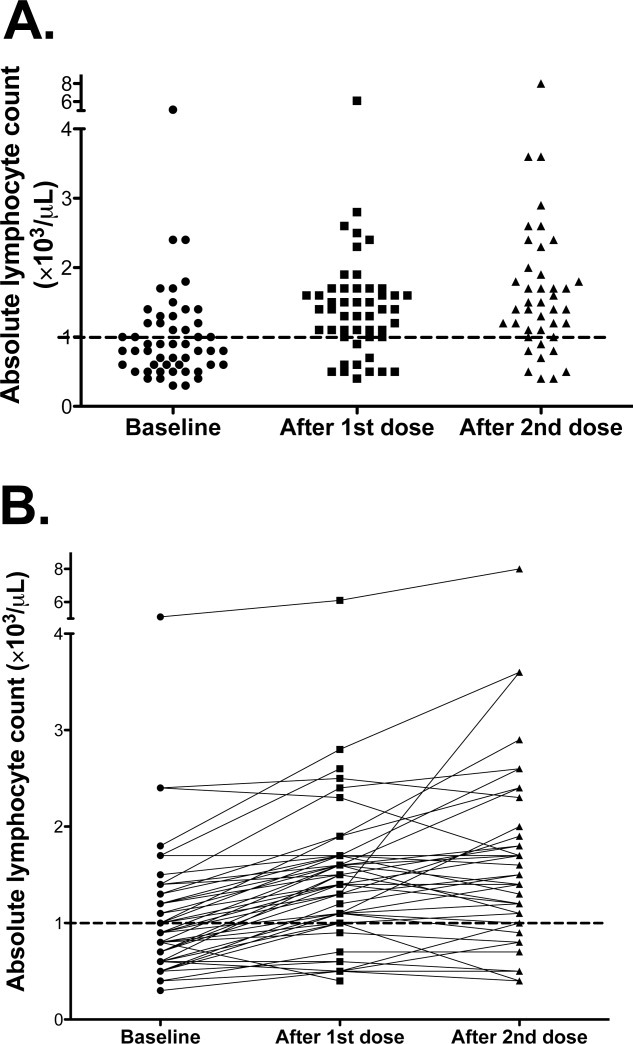
Changes in the absolute lymphocyte count (ALC) with ipilimumab therapy are shown. (A) The ALC of all patients at baseline and after 1 and 2 doses of ipilimumab is shown. (B) The change in ALC for each patient with therapy is shown.

**Figure 3:**
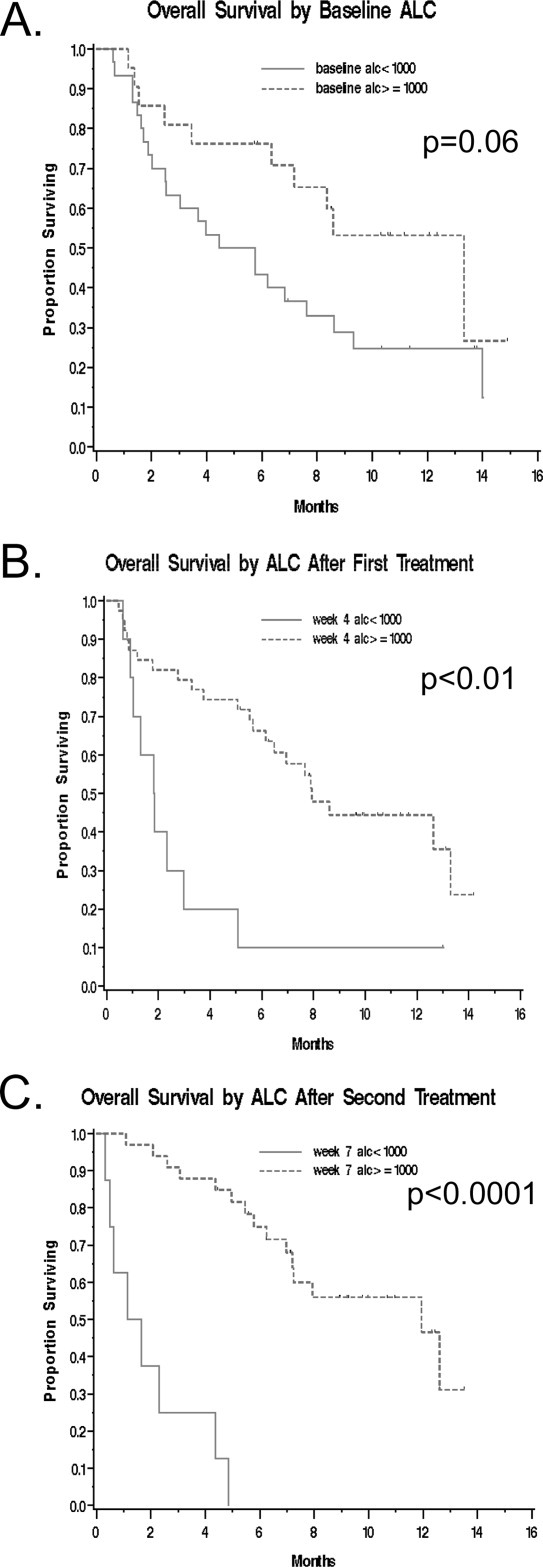
Kaplan-Meier survival curves are shown stratified by the absolute lymphocyte count (ALC) at (A) baseline and after (B) the first and (C) second ipilimumab doses.

We sought to correlate ALC at different early time points with the rate of clinical benefit at Week 24 and OS. ALC values at different time points are shown in Figure 2. We stratified patients based on a cutoff of ≥1000/μL (high ALC) versus <1000 cells/μL (low ALC). Kaplan-Meier survival curves based on the ALCs at baseline and after 1 and 2 ipilimumab doses, respectively, are shown in Figure 3.

When patients were stratified based on their baseline ALC, there was a nonsignificant trend toward an increased rate of clinical benefit at Week 24 for patients with a high versus low ALC (10 of 21 [48%] patients vs 7 of 30 [23%]; *P* = .07). There was also a borderline significant trend toward improved OS for the high ALC group (median OS, 13.3 months vs 5.1 months; *P* = .06). This trend remained after adjusting for baseline LDH (*P* = .06). The 6-month and 12-month OS were 76% versus 43% and 53% versus 25%, respectively, when stratified by high versus low ALC (Fig. .3A).

When patients were stratified by their ALC after 1 ipilimumab dose (obtained 3 weeks later on the day of their planned second ipilimumab dose), there was a nonsignificant trend toward increased clinical benefit at Week 24 for high versus low ALC patients (16 of 39 [41%] patients vs 1 of 10 [10%]; *P* = .07). Patients with a high ALC after 1 ipilimumab dose did have significantly improved OS (median OS, 7.9 months vs 1.8 months; *P* < .01). This trend remained after adjusting for baseline LDH (*P* < .01). The 6-month and 12-month OS were 66% versus 10% and 44% versus 10%, respectively, by high versus low ALC (Fig. .3B).

Finally, we stratified patients by their ALC after 2 ipilimumab doses (obtained 3 weeks later on the day of their planned third ipilimumab dose). Patients with a high ALC had a significantly higher clinical benefit rate at Week 24 compared with those with a low ALC (17 of 33 patients [51%] vs 0 of 8; *P* < .01) as well as improved OS (median OS, 11.9 months vs 1.4 months; *P* < .0001). This trend remained after adjusting for baseline LDH (*P* < .0001). The 6-month and 12-month OS rates were 75% versus 0% and 47% versus 0%, respectively, by high versus low ALC (Fig. .3C).

## DISCUSSION

The results of this trial of compassionate use ipilimumab at MSKCC are largely consistent with the results presented in abstract form for several other phase 2 trials of similar doses/schedules of ipilimumab.^20–22^ Specifically, our objective RR of 12% (as adjudicated by the proposed immune-related response criteria) is very comparable to the objective RRs of 5.8% to 15.8% adjudicated by standard modified WHO criteria that are reported in other phase 2 trials. These trials also reported CR + PR + SD rates of 27.1% to 35.1%, similar to our immune-related CR + immune-related PR + immune-related SD rate of 41%. Finally, grade 3 to 4 immune-related adverse events were noted in 20% to 38.6% of patients in these trials, consistent with the rate of 29% we describe here.

In addition, the results of a phase 1 to 2 trial of 2 doses and schedules of tremelimumab, another anti–CTLA-4 antibody, were recently published.^23^ This trial reported an objective RR of 9% by Response Evaluation Criteria in Solid Tumors criteria, a CR + PR + SD rate of 39% to 41%, and a grade 3 to 4 toxicity rate of 13% to 27%. Median time to progression was about 1.9 months, with median OS of 9.97 to 11.53 months. With the exception of the longer OS reported in this trial, which was likely because of the enrollment of only treatment-naive patients with relatively low LDH, and despite the caveats of comparing different phase 2 trials, these results are otherwise strikingly similar to ours. Certainly, the median OS of 7.2 months that we report in our heavily pretreated patients also compares favorably to standard first-line therapies for melanoma, such as temozolomide or dacarbazine.^24^

In this study, results were reported according to immune-related response criteria because these were used for clinical decision making. When we also evaluated responses using traditional modified WHO criteria, we noted strong agreement between both criteria. As might be expected, the only discrepancies occurred when 2 patients who were adjudicated to have immune-related SD would have experienced PD by the modified WHO criteria because of the development of new metastatic lesions (which increased the total tumor burden used in the immune-related criteria by <25%). As such, there was no change in the objective RR or best response rate of SD or PD. There was a slight difference in the Week 24 CR + PR + SD rate of 30% (15 of 51 patients) versus the Week 24 immune-related CR + immune-related PR + immune-related SD rate of 33% (17 of 51 patients), as well as a small change in the median PFS by modified WHO versus immune-related criteria (2.5 vs 2.6 months, respectively).

Our results suggest that patients who develop grade 3 to 4 immune-related adverse events are more likely to experience clinical benefit at Week 24 compared with those with no or mild immune-mediated toxicity. These results are consistent with prior reports^25–27^ and are also biologically consistent with the belief that CTLA-4 blockade acts in a nonspecific fashion to de-repress the immune system, resulting in the frequent but not absolute co-occurrence of antitumor effect and autoimmune-like toxicity. Of note, the median number of ipilimumab treatments that patients with grade 3 to 4 immune-related adverse events received (which was 4) was identical to the median number of treatments patients with grade ≤2 immune-related adverse events received. The comparable duration of therapy suggests that the increased grade 3 to 4 immune-related adverse event rate in patients with clinical benefit was not likely to be because of increased exposure to ipilimumab.

Remarkably, our analysis shows that the ALC correlates strongly both with clinical benefit and OS. The ALCs obtained at baseline and after 1 ipilimumab treatment appear to be prognostic, but these early ALCs may be more of a reflection of the extent of prior therapy and tumor burden. However, the suggestion that a low baseline ALC may be associated with poorer OS does have implications for the sequencing of therapy in future trials that combine ipilimumab with chemotherapy.

The ALC obtained after the second ipilimumab dose appears to be a particularly informative biomarker. None of 8 patients with an ALC <1000 cells/μL experienced clinical benefit at Week 24; these patients also had statistically and clinically significantly inferior OS compared with patients with an ALC ≥1000 cells/μL. There was a difference in objective RR between both groups (18% vs 0%), but this was not statistically significant (*P* = .33), possibly because of the small number of objective responses that were noted in this trial.

The relatively small number of patients in this trial precluded us from performing a detailed multivariate analysis, although the ALC remained a significant prognostic marker when we controlled for baseline LDH. Nevertheless, the observation that other prognostic factors (number of prior therapies and the primary site of the tumor) were not correlated with OS suggests that the ALC does represent an independent biomarker.

There is a strong biological rationale that the ALC value is correlated with benefit from ipilimumab because it directly blocks CTLA-4 expressed on various lymphocyte populations. Presumably, a threshold ALC of 1000 cells/μL reflects the underlying capacity of the immune system to be adequately activated by ipilimumab to mediate clinically meaningful antitumor effects. Our observation also complements recent data indicating that the rate of change of ALC with ipilimumab therapy is positively associated with the CR + PR + SD rate.^28^ In particular, the observation from this larger data set that patients whose ALCs drop after initiation of ipilimumab have a 0% CR + PR + SD rate is consistent with our observation that a minimum ALC level may be required for patients to derive clinical benefit from ipilimumab.

Overall, these data have strong implications for clinical practice, as they suggest that the approximately 20% of patients with an ALC <1000 cells/μL will not benefit from additional ipilimumab therapy. Such patients could be spared the potential toxicity of further therapy and could be switched to an alternative treatment plan.

In conclusion, this report of patients with advanced melanoma treated at MSKCC in a trial of compassionate use ipilimumab is consistent with other phase 2 evaluations of anti–CTLA-4 antibodies. Furthermore, our results suggest a single ALC measurement obtained after 2 ipilimumab treatments is strongly predictive of clinical response to ipilimumab. A larger retrospective multivariate review and prospective validation of this biomarker are to be strongly considered, as they have the potential to identify patients who are unlikely to benefit from ipilimumab therapy, while benefiting those who are likely to benefit.
